# Granulocyte-Derived Extracellular Vesicles Activate Monocytes and Are Associated With Mortality in Intensive Care Unit Patients

**DOI:** 10.3389/fimmu.2018.00956

**Published:** 2018-05-08

**Authors:** Ali Danesh, Heather C. Inglis, Mohamed Abdel-Mohsen, Xutao Deng, Avril Adelman, Kenneth B. Schechtman, John W. Heitman, Ryan Vilardi, Avani Shah, Sheila M. Keating, Mitchell J. Cohen, Evan S. Jacobs, Satish K. Pillai, Jacques Lacroix, Philip C. Spinella, Philip J. Norris

**Affiliations:** ^1^Blood Systems Research Institute, San Francisco, CA, United States; ^2^Department of Laboratory Medicine, University of California, San Francisco, San Francisco, CA, United States; ^3^Division of Biostatistics, Washington University School of Medicine in St. Louis, St. Louis, MO, United States; ^4^Department of Medicine, Washington University School of Medicine in St. Louis, St. Louis, MO, United States; ^5^Department of Surgery, University of California, San Francisco, San Francisco, CA, United States; ^6^Centre Hospitalier Universitaire (CHU) Sainte-Justine, Université de Montréal, Montreal, QC, Canada; ^7^Department of Pediatrics, Washington University School of Medicine in St. Louis, St. Louis, MO, United States; ^8^Department of Medicine, University of California, San Francisco, San Francisco, CA, United States

**Keywords:** extracellular vesicles, monocytes, granulocytes, exosomes, microvesicles, mortality, intensive care unit, receptor

## Abstract

To understand how extracellular vesicle (EV) subtypes differentially activate monocytes, a series of *in vitro* studies were performed. We found that plasma-EVs biased monocytes toward an M1 profile. Culturing monocytes with granulocyte-, monocyte-, and endothelial-EVs induced several pro-inflammatory cytokines. By contrast, platelet-EVs induced TGF-β and GM-CSF, and red blood cell (RBC)-EVs did not activate monocytes *in vitro*. The scavenger receptor CD36 was important for binding of RBC-EVs to monocytes, while blockade of CD36, CD163, CD206, TLR1, TLR2, and TLR4 did not affect binding of plasma-EVs to monocytes *in vitro*. To identify mortality risk factors, multiple soluble factors and EV subtypes were measured in patients’ plasma at intensive care unit admission. Of 43 coagulation factors and cytokines measured, two were significantly associated with mortality, tissue plasminogen activator and cystatin C. Of 14 cellular markers quantified on EVs, 4 were early predictors of mortality, including the granulocyte marker CD66b. In conclusion, granulocyte-EVs have potent pro-inflammatory effects on monocytes *in vitro*. Furthermore, correlation of early granulocyte-EV levels with mortality in critically ill patients provides a potential target for intervention in management of the pro-inflammatory cascade associated with critical illness.

## Introduction

Extracellular vesicles (EVs) are double membrane vesicles that can be released from virtually all cell types under physiological and pathological conditions and may be detected in blood and other body fluids (1, 2). The role of EVs in cell–cell communication and immunity is a new area in biology and medicine, and immunosuppressive and immunostimulatory roles have been attributed to EVs (3–6). We previously showed that EVs from stored leukoreduced blood are heterogeneous and originate from multiple cell types. We observed that monocytes bind to and engulf EVs, and T cell response modulation by EVs is indirect and mediated *via* monocyte activation (7). Red blood cell (RBC)-EVs have been described as immunosuppressive or immunostimulatory in separate studies (8, 9). The literature is also conflicting on the stimulatory vs. suppressive effect of platelet-EVs on monocytes and macrophages (10, 11).

Understanding how EV subtypes interact with immune cells would better allow their manipulation in disease states, as several mechanisms of uptake have been described for EVs. EV surface proteins can play an important role in EV uptake, as treatment of EVs with proteinase K decreases the uptake of EVs by ovarian cancer cells (12). Phagocytosis, clathrin-mediated endocytosis, caveolin-mediated endocytosis, and membrane fusion are suggested mechanisms for EV uptake (13). It is believed that adhesion molecules, integrins, and lectins play a role in EV uptake (13–15). Proteoglycans such as heparin sulfate may also play a role in EV uptake, as treatment of cells with a heparin sulfate mimetic reduces EV uptake (16). The role of TLRs in EV uptake has also been studied, and the data in the literature on TLRs are conflicting (17, 18). In general, EV uptake can involve several receptors (12, 19–24). The role of scavenger receptors in EV uptake is not well studied, but it has been shown that endothelial-EVs bind to the scavenger receptor CD36 on platelets and contribute to thrombosis in mice (25).

Increased levels of particular EV subtypes have been associated with specific diseases, and EV subtypes may serve as novel biomarkers. The plasma level of CD31^+^ EVs is associated with increased risk of cardiovascular death (26). Tissue factor (CD142)-positive EVs derived from endothelial cells and monocytes in sickle cell disease contribute to thrombin generation and coagulation (27). In a study of critically ill patients, the ratio of platelet-EVs to platelet count was associated with mortality, primarily driven by an inverse relationship between platelet count and mortality (28). In critically ill burn patients, white blood cell (WBC)- and granulocyte-EVs at intensive care unit (ICU) admission are associated with subsequent mortality (29).

Using RNA sequencing and global transcriptomic analyses, here we show that plasma-EVs bias primary monocytes toward an M1 profile, which leads to generation of a dominant inflammatory response. We also show that whether EVs induce pro- or anti-inflammatory responses in monocytes depends on their cell of origin. Finally, we demonstrate that a group of scavenger receptors were regulated in monocytes stimulated with EVs, and that RBC-EVs bind monocytes at least in part *via* the scavenger receptor CD36. We enrolled a subset of 100 critically ill subjects from three of the clinical sites participating in the Age of BLood Evaluation (ABLE) trial and measured a broad array of immune and coagulation parameters to determine if the age of blood transfused affected these parameters, and secondarily whether any of the parameters predicted subsequent mortality (30). We showed that in addition to cystatin C and tissue plasminogen activator (TPA), EVs expressing CD66b (granulocyte), CD15 (granulocyte and monocyte), CD11b (adhesion molecule), and CD62P (activated platelets and endothelial cells) are early predictors of mortality in ICU patients.

## Materials and Methods

### Study Samples

For *in vitro* experiments Trima filters (discarded byproducts of platelet apheresis) were used to generate large stocks of stored peripheral blood mononuclear cells (PBMCs). Fresh blood from six healthy donors was used for isolation of granulocytes to generate pure granulocyte-EVs. To purify RBC- and platelet-EVs, RBC units and platelet units were washed by automation and stored for 21 and 5 days, respectively. All filters and units were de-identified and acquired from Blood Centers of the Pacific (BCP). All study protocols were approved by the University of California, San Francisco Committees on Human Research.

Samples from the ABLE study were used for *ex vivo* experiments. ABLE was a multicenter, randomized, controlled clinical trial that studied the effect of RBC unit storage time in 1,430 critically ill patients who received RBC transfusion. PBMC samples from a subset of 100 patients in the ABLE trial were collected pre-transfusion and on days 2, 6, 28, and 180 post-transfusion. ABLE sites participating in this study included The Ottawa Hospital (General and Civic campuses) and the Institut de Cardiologie et de Pneumologie de Québec, Université Laval. All patients from the ABLE trial were eligible to participate, with the exception of those with history of bone marrow transplantation. Plasma samples were used for measurement of EVs, cytokines, growth factors, and coagulation factors. In addition, clinical data were collected in the ABLE trial, including mortality and multiorgan dysfunction syndrome score. Samples were collected under informed consent and IRB approval in accordance with the Declaration of Helsinki. A group of 48 healthy control subjects was enrolled at Blood Systems Research Institute, with a blood sample collected at a single time point for analysis of EV subtypes in peripheral blood.

### Sample Processing

Plasma-EVs were isolated from ACD-treated blood using differential centrifugation. Plasma was separated at 1,000 *g* from cells and spun at 13,000 *g* to make platelet-free plasma (PFP). Six mL of PFP were added to 30 mL phosphate-buffered saline and spun for 1 h at 100,000 *g*. EV pellets were resuspended in 1 mL RPMI and stored at −80°C. Purified EVs were used for functional experiments.

For measurement of EV subtypes in peripheral blood, whole blood from 48 donors was collected in a citrate tube. Tubes were centrifuged at 2,500 *g*, and plasma was stored in 0.5 mL aliquots at −80°C until testing. Whole blood was collected in EDTA tubes from ABLE study subjects on day 0 (before transfusion) and on days 2, 6, and 28 after the first RBC transfusion. Collection tubes were centrifuged at 1,000 *g* to separate cells from plasma, and plasma was centrifuged at 13,000 *g* for removal of platelets and large fragments of cells. Aliquots of 0.5 mL PFP were stored at −80°C until testing.

### Generation of Pure EV Subtypes Based on Their Cell of Origin

Pure RBC-, platelet-, monocyte-, granulocyte-, and endothelial-EVs were prepared for functional experiments. Monocytes were isolated from PBMCs of healthy donors by double negative selection using an EasySep Human Monocyte Enrichment Without CD16 Depletion Kit (Stemcell Technologies), and they were cultured at 1 million cells/mL for 2 days to generate monocyte-EVs in the culture supernatant. Whole blood was treated with HetaSep (Stemcell Technologies) to sediment RBCs and isolate leukocytes. Granulocytes were isolated from leukocytes by double negative selection using an EasySep Human Pan-Granulocyte Isolation Kit (Stemcell Technologies) and were cultured at 1 million cells/mL for 24 h to generate granulocyte-EVs. Washed leukoreduced RBC units were stored for 21 days in CP2D plus AS3 storage solution at 4°C to generate pure RBC-EVs. Platelet units were washed and stored for 5 days on a shaker at 25°C to generate platelet-EVs. Human umbilical vein endothelial cells (University of California, San Francisco Cell Culture Facility) were cultured to 90% confluence for 1 week to generate endothelial-EVs. EV subtypes were isolated from monocyte, granulocyte, and endothelial cell culture supernatants, and from stored RBC units and stored platelets by differential centrifugation as described earlier, followed by storage at −80°C.

### Characterization of EVs

To characterize EVs from patients, PFP samples were stained and acquired as previously described (31) using 14 different fluorochrome-conjugated monoclonal antibodies in three separate panels, including CD235a-FITC, CD62P-APC, CD3-PerCP/Cy5.5, CD19-Alexa/700, CD28-FITC, CD16-V450, CD62L-APC, CD11b-PE/Cy7, CD66-PE (BioLegend), CD15-FITC (ExAlpha), CD152-APC, CD14-APC/Cy7, CD108a-PE, and CD41a-PerCP/Cy5.5 (BD Biosciences). In normal donor samples CD142-PE (BioLegend) and CD154-APC (BD Biosciences) were substituted for CD14 and CD152, respectively. To reduce the background staining and as the size of small EVs fall below the detection limit of flow cytometer, stained EVs were diluted in PBS and were centrifuged for 5 min at 500 *g* using 0.22 µm Ultrafree MC-GV Centrifugal Filter Units (Millipore). Flow through (small EVs and unbound antibodies) was discarded, and stained large EVs were harvested in PBS from the top of the filter. Data were acquired using an LSR II flow cytometer (BD Biosciences). FSC/SSC voltages were set to the highest values that excluded the majority of background noise (i.e., just below the voltage threshold at which event rate surpassed 5 events/s while running a tube of PBS alone). Typically, this threshold occurred at FSC and SSC voltages of around 500–600 and 300–350, respectively. Gates were set using beads sized 100, 200, 240, 500, and 1,000 nm (Megamix-Plus SSC; BioCytex), and EVs were collected from the threshold to the 1,000 nm gate based on SSC. Analysis was performed using FlowJo 7.6.5 software (Tree Star).

### Isolation of PBMCs and Purification of Monocytes

Whole blood or leukocytes trapped in TRIMA filters were overlaid on Ficoll-Paque (Sigma-Aldrich) and centrifuged for 30 min at 600 *g*. PBMC layers were harvested followed by washing and cryopreservation in fetal bovine serum (FBS) containing 10% DMSO. Isolation of monocytes from PBMCs was performed by double negative selection from PBMCs using the EasySep Human Monocyte Enrichment Without CD16 Depletion Kit (Stemcell Technologies).

### Stimulation of Monocytes With EVs

Extracellular vesicles were counted using Trucount Absolute Counting Tubes (BD Biosciences). To each Trucount™ tube, 50 µL sample and 350 µL PBS were added and samples were read immediately on the flow cytometer. EV concentrations were calculated using the following equation:

EVs/μL=[EV region events/bead region events]×[Trucount™ beads/μL of sample added].

One million monocytes were cultured in 1 mL of 10% inactivated exosome-free FBS (SBI) in RPMI (containing 10 mM *N*-2-hydroxyethylpiperazine-*N*′-2-ethanesulfonic acid, 100 U/mL penicillin G, and 100 mg/mL streptomycin) in the presence or absence of one million plasma-EVs in a 5% CO_2_ incubator at 37°C for 1, 3, and 24 h. Each experiment was performed in duplicate wells, and cell culture supernatants (0.3 mL) were harvested for cytokine assays and cells were added to 0.7 mL QIAzol Lysis Reagent (Qiagen) for total RNA isolation. For experiments using individual EV subtypes, 1 million monocytes were cultured with 1 million EVs of a given subtype (RBC-, platelet-, monocyte-, endothelial-, and granulocyte-EVs) for 24 h, and supernatants were harvested for cytokine assays. For some experiments EVs were fractionated by size. Briefly, EVs were centrifuged at 500 *g* using 0.22 µm Ultrafree MC-GV Centrifugal Filter Units (Millipore) to separate small and large EVs (enriched for exosomes and MVs, respectively). The small EVs (<220 nm) were collected in the flow through, and the large EVs (>220 nm) were recovered from the top of the filter. Each fraction was incubated with monocytes for 16 h followed by permeabilization of cells using Cytofix/Cytoperm Kit (BD Biosciences) and intracellular staining for TNF-α (-V421 labeled, BioLegend).

### Gene Expression Profiling With High Throughput Sequencing

Total RNA from monocytes, EVs, and PBMC positive controls was extracted using the miRNeasy Mini Kit (Qiagen) with the optional on-column DNAse treatment step. RNA was quantified using a NanoDrop ND-1000 Spectrophotometer (NanoDrop Technologies) and integrity was assessed using an Agilent 2100 Bioanalyzer (Agilent Technologies). For RNA-Seq experiments cDNA was generated using the Illumina TruSeq Stranded mRNA Library Prep Kit (Illumina Technologies), and 400 ng of total RNA was used as input. Single-read sequencing was performed using the Illumina HiSeq 2000 instrument to obtain 30–50 million single reads of 51 nucleotides. RNA-Seq data were preprocessed by adaptor trimming and low quality 3′-tail trimming (Phred > 20). The preprocessed reads were mapped using Tophat to the reference genome hg19. Gene level expression quantification in FPKM (Fragments Per Kilobase of transcript per Million mapped reads) was calculated using Cufflinks suite including Cufflinks, Cuffmerge, Cuffquant, and Cuffnorm. Significant changes in transcript expression were quantified by *t*-test, which were adjusted by false discovery rate (FDR < 0.05). Gene annotations and GO terms were extracted from BioMart using the Bioconductor/biomaRt package.

### Intracellular Cytokine Assays and TLR Blocking Antibodies

Intracellular staining of monocytes for detection of TNF-α was performed after stimulation of 1 million/mL monocytes with EVs as previously described (7). To test the efficacy of anti-TLR neutralizing antibodies, one million PBMCs were treated with 1 µg/mL TLR1, TLR2, or TLR4 antibodies for 1 h, and then stimulated with TLR agonists (InvivoGen) for 16 h. Synthetic tripalmitoylated lipopeptide Pam3CysSerLys4 (Pam3CSK4, 20 ng/mL) was used as a TLR1 and TLR2 agonist, and LPS (5 ng/mL) was used as a TLR4 agonist. Finally, cells were stained with CD14-APC/Cy7 antibody (BioLegend) before permeabilization, then washed and stained for TNF-α, and run on the flow cytometer.

### Measurement of Cytokines in Supernatant of Monocytes Stimulated With EVs

A Milliplex MAP Kit (Millipore) was used to measure the level of 13 cytokines and growth factors in culture supernatants of monocytes stimulated with plasma-EVs (GM-CSF, IFNγ, IL-10, IL-12p70, IL-13, IL-1β, IL-2, IL-4, IL-5, IL-6, IL-7, IL-8, and TNF-α). A second Milliplex MAP kit from the same manufacturer was used to measure the level of 12 cytokines in supernatants of monocytes stimulated with EV subtypes (IFNγ, IL-10, sCD40L, IL-1RA, IL-1α, IL-1β, IL-6, MIP-1α, TNF-α, MIG, GM-CSF, and TGF-β). A Bio-Plex 200 instrument (Bio-Rad) was used for data acquisition.

### Cell Surface Expression of Scavenger Receptors and TLRs

Cell surface expression of scavenger receptors CD36, CD163, and CD206, and cell surface expression of TLR1, TLR2 and TLR4 on monocytes were assessed by staining PBMCs with CD14-PerCP/Cy5.5, CD36-PE, CD163-APC, CD206-Alexa Fluor 488, TLR2-PE, TLR4-APC (BioLegend), and TLR1-FITC (Invivogen) fluorochrome-conjugated antibodies according to the manufacturers’ instructions. Samples were fixed in 2% paraformaldehyde solution and were run on the flow cytometer. Percent expression of scavenger receptors and MFI of TLRs were measured after gating on CD14^+^ monocytes.

### EV–Monocyte Binding Assay

Plasma-EVs, aged RBC-EVs, or EV subtypes were stained with PKH26 Red Fluorescent Cell Linker Dye (Sigma-Aldrich). EVs were washed twice with 10% exosome-free FBS in RPMI to quench the unbound dye. Recombinant annexin V (BD Biosciences) or functional grade blocking monoclonal antibodies against phosphatidylserine (PS) (Millipore), CD36 (Stemcell Technologies), CD163, CD206 (BioLegend), TLR1 (Invivogen), TLR2, and TLR4 (BioLegend) were used at multiple concentrations (0.01–2.0 µg/mL) to block EV–monocyte binding. In some experiments, EVs were incubated with annexin V or anti-PS antibody. In other experiments PBMCs were incubated with monoclonal antibodies against CD36, CD163, CD206, TLR1, TLR2, or TLR4 in a 5% CO_2_ incubator at 37°C for 1 h. PBMCs (500,000) were cultured with EVs, in a final volume of 0.5 mL for 24 h. PBMCs were stained with CD14-PerCP/Cy5.5 (BioLegend) and were fixed in 2% paraformaldehyde solution. PBMCs were subject to flow cytometry, and percent binding of monocytes to EVs was measured by gating on CD14^+^ cells.

### Measurement of Cytokines and Coagulation Factors

PFP from 100 ABLE study subjects was analyzed for levels of immune and coagulation markers. To determine the inflammatory and coagulation profile of PFP, a total of 43 different markers were measured, including 16 coagulation factors: prothrombin time, partial thromboplastin time, D-dimer concentration, factor II, factor V, factor VII, factor VIII/40, factor IX/20, factor X, antithrombin III, protein C, fibrinogen concentration, thrombomodulin, endothelial cell protein C receptor, TPA, and plasminogen activator inhibitor type-1 (PAI-1). The markers of coagulation were analyzed on a Stago or Dade Behring-Siemens device. Coagulation factors Va, VIIIa, VII, as well as antithrombin III, prothrombin time, partial thromboplastin time, TPA, D-dimer, and protein C were measured on a Diagnostica Stago™ coagulation analyzer. Concentrations of prothrombin fragments 1 + 2, soluble thrombomodulin, PAI-1, soluble endothelial protein C receptor, and cytokines were measured using commercially available ELISA kits. In addition, 27 cytokines were measured using Milliplex MAP kit (Millipore): GM-CSF, IL-12p70, IL-17A, IL-1β, IL-2, IL-21, IL-23, IL-6, IL-7, IL-8, ITAC, MIP-1α, MIP-1β, TNF-α, EGF, FGF, IFN-γ, IP-10, VEGF, β2-microglobulin, cystatin C, myeloperoxidase, PAI-1, PDGF-AB/BB, RANTES, sICAM-1, and sVCAM–1. A Bio-Plex 200 instrument (Bio-Rad) was used for cytokines data acquisition.

### Data Analysis

Supervised gene analysis was performed on all genes that were mapped by high throughput sequencing and used in this paper. FDRs were computed using the Benjamini–Hochberg procedure to adjust for multiple comparisons in the RNA-Seq data. The heat maps were generated using standardized *Z*-scores. GraphPad Prism v.6 was used for ANOVA and *t*-test analyses as noted in the figure legends. The significance of predictors of mortality was based on Cox regression analyses. The distribution of the data was analyzed, and for analytes with non-normally distributed data, values were log-transformed before analysis.

## Results

### EV Exposure Initiates an M1 Phenotype Gene Expression Program

**Figure 1:**
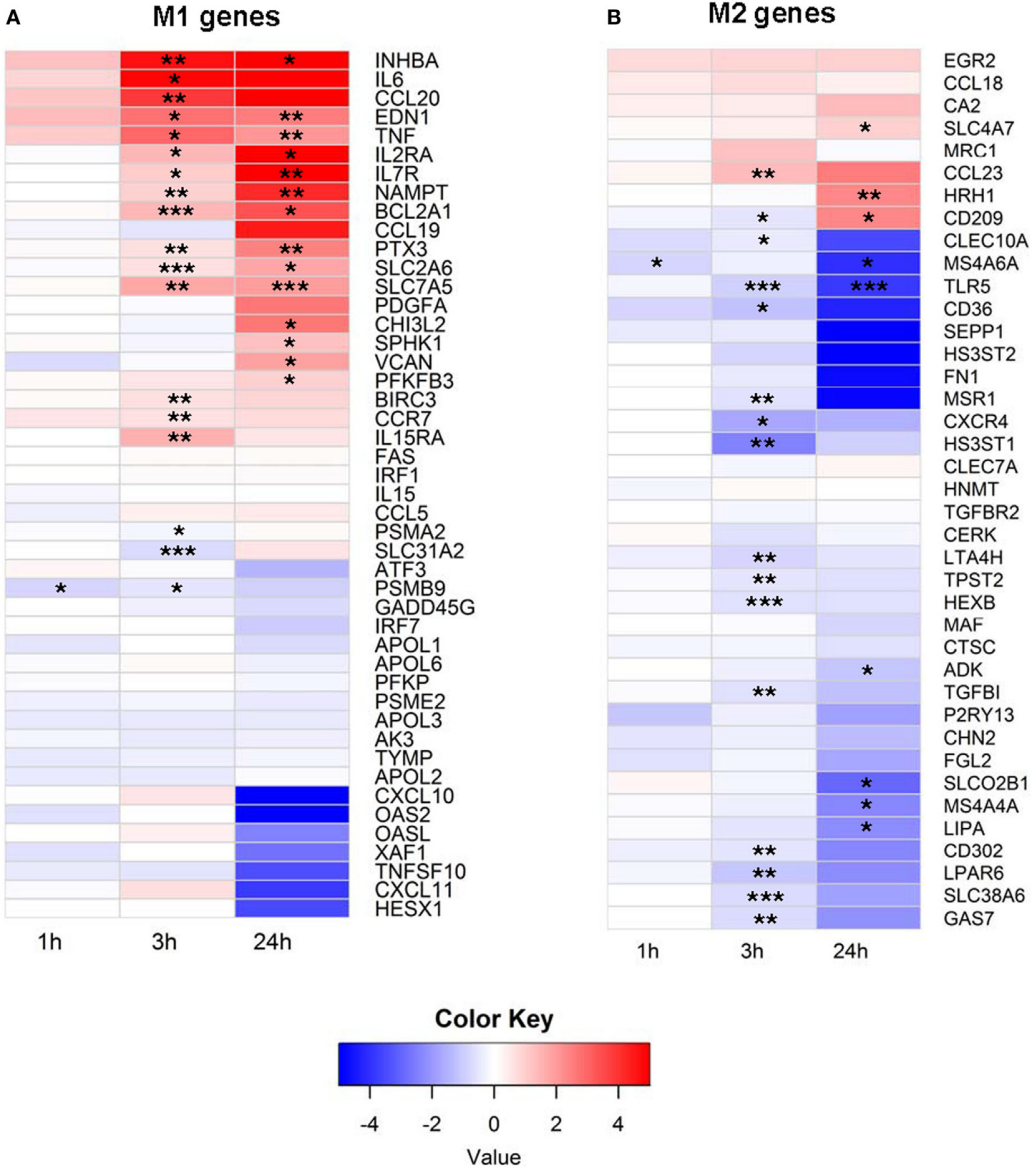
**(A)** M1 and **(B)** M2 mRNA expression profile of extracellular vesicle (EV)-exposed monocytes. Monocytes were purified from peripheral blood mononuclear cells of five healthy donors and were cultured unstimulated or stimulated with plasma-EVs from five other healthy donors for 0, 1, 3, and 24 h. mRNA expression was determined by RNA-Seq and analyzed longitudinally for a panel of previously described M1 and M2-associated genes. Results for EV-incubated conditions were normalized to matched, unstimulated conditions at each time point and log_2_-transformed (*p < 0.05, **p < 0.01, and ***p < 0.001).

To better understand how EVs affect human immune cells, we performed transcriptomic analyses using RNA-Seq on monocytes at baseline and longitudinally after exposure to EVs derived from PFP. It has been shown that monocytes and monocyte-derived macrophages internalize EVs, which leads to their activation (11, 32, 33). Our prior work demonstrated that monocytes ingest EVs found in stored RBC units (7), therefore we focused our studies on this population of immune cells. It has been shown that several genes are expressed differentially during the process of monocyte polarization to M1 and M2 profiles, and that the M1 phenotype has pro-inflammatory effects, while the M2 phenotype possesses anti-inflammatory properties (34). We analyzed 53 genes that have been described as associated with the M1 profile and 43 genes associated with the M2 profile (34). From M1-associated genes, the mRNA of 19 genes were significantly upregulated at 3 and/or 24 h, including *NAMPT, IL15RA, VCAN, CHI3L2, IL7R, IL2RA, PTX3, SLC2A6, BIRC3, SPHK1, TNF, EDN1, BCL2A1, CCR7, CCL20, IL6, INHBA, PFKFB3*, and *SLC7A5* (Figure 1A). Three genes were downregulated at 1 and/or 3 h, including *SLC31A2, PSMB9*, and *PSAM2*. Analysis of M2 genes showed that the majority of M2-biasing genes with significant changes after EV exposure were downregulated (Figure 1B). We found that mRNA of 19 genes were downregulated, most notably at 3 h post EV exposure, including *MSR1, CXCR4, CD302, GAS7, TPST2, CD36, MS4A6A, LTA4H, TLR5, SLC38A6, SLEC10A, LIPA, MS4A4A, SLCO2B1, LPAR6, TGFBI, ADK, HS3ST1*, and *HEXB*. Only four M2-associated genes were upregulated, including *SLC4A7, CD209, CCL23*, and *HRH1*. In summary, exposure of primary monocytes to EVs from plasma of healthy donors led to predominant upregulation of M1-associated genes (19 up and 3 down) and downregulation of M2-associated genes (4 up and 19 down), and the different regulation pattern of M1 and M2-associated genes was significant (*p* < 0.0001, Fisher’s exact test).

### Effector Molecules Induced in Monocytes Exposed to EVs

**Figure 2:**
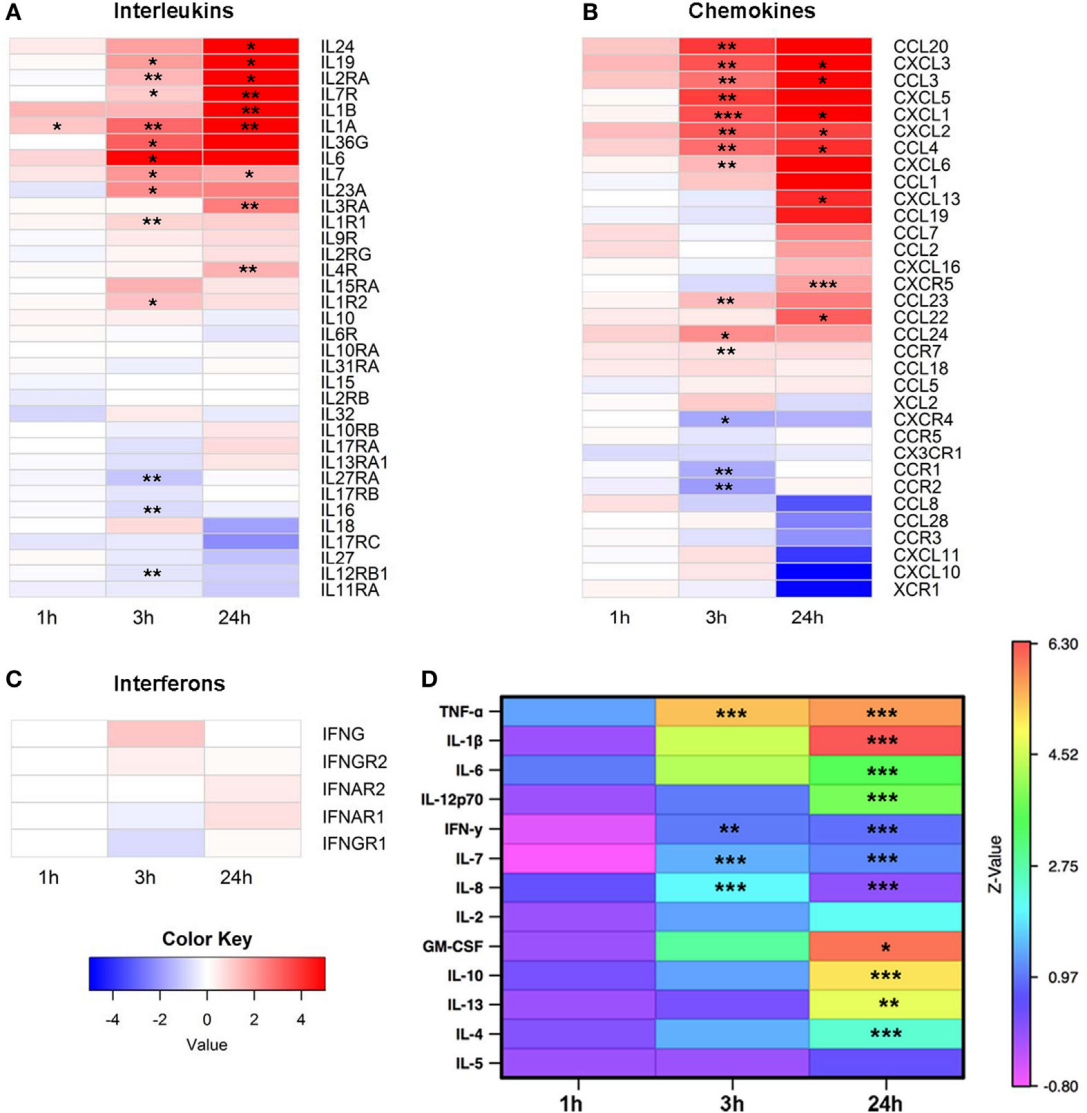
Expression of cytokines, chemokines, and growth factors. Monocytes were purified from peripheral blood mononuclear cells of five healthy donors and were cultured unstimulated or stimulated with plasma-EVs from five other healthy donors for 0, 1, 3, and 24 h. Supervised gene analysis was performed, and significant changes in transcript expression quantified by *t*-test, which were adjusted by false discovery rate (<0.05). **(A)** Of the panel of genes for interleukins or their receptors, expression of 14 was significantly upregulated and of 3 was downregulated at 3 and/or 24 h in monocytes stimulated with EVs compared with paired unstimulated samples. **(B)** Of the chemokines and their receptors, expression of 14 was significantly upregulated and of 3 was downregulated at 3 and/or 24 h. **(C)** mRNA expression of most interferons was not detectable, and of the detectable messages none was significantly different from unstimulated monocytes. **(D)** Supernatants were collected from the same cultures used for mRNA expression analysis. Results for stimulated conditions were normalized to unstimulated data and log_2_-transformed to show log-fold increase after stimulation (*p < 0.05, **p < 0.01, and ***p < 0.001).

The RNA-Seq data were next analyzed to examine expression of cytokines and growth factors in more detail. From 36 known interleukins, message for 27 was detectable by high throughput sequencing. The mRNA expression of 14 interleukins and interleukin receptors was significantly upregulated at 3 and/or 24 h (Figure 2A). We next looked at the gene expression of chemokines and found 24 mRNA transcripts that were detectable. Expression of 17 chemokines changed significantly, with the majority upregulated at 3 and/or 24 h (Figure 2B). In contrast to interleukins and chemokines, most interferon transcripts were not detectable, and of the five that were, none showed significant changes after EV exposure (Figure 2C).

To determine whether the changes in cytokine mRNA levels measured after monocyte exposure to EVs translated to changes in protein levels, the supernatants from the same stimulated monocytes were tested using a multiplex cytokine assay. The levels of nine pro-inflammatory and four anti-inflammatory cytokines were measured at 0, 1, 3, and 24 h intervals in the supernatant of monocytes that were unstimulated or incubated with EVs isolated from normal donor plasma, and values were reported as a ratio of EV-exposed to unstimulated conditions at each time point (Figure 2D). Of the cytokines in the panel, mRNA levels of *TNF* and *IL7* were elevated at 3 and 24 h after EV exposure, and mRNA from *IL1B* was elevated at 24 h. Protein levels of all these analytes were significantly elevated and concordant with the mRNA expression data, with the exception that *IL6* mRNA was significantly elevated at 3 h while the elevation in IL-6 protein did not reach significance until 24 h. In addition, there were two cytokines with elevated protein levels at 3 h (IFN-γ and IL-8) and seven cytokines or growth factors with elevated protein levels at 24 h that were either not significantly elevated or not detected in the mRNA analysis (*IL-12p70*, IFN-γ, *IL-8, GM-CSF, IL-10, IL-13*, and *IL-4*). Examination of the mRNA and protein expression data of four representative cytokines revealed similar patterns between mRNA and protein expression for TNF-α, IL-1β, and IL-6, while increases in IL-10 were detectable at the protein but not mRNA expression level (Figure S1 in Supplementary Material). To determine whether EV-derived mRNA contributed to the signal in the RNA-Seq experiments, RNA was quantified in the monocyte preparations as well as separate PBMC, EV, and blank well conditions. Monocyte and PBMC controls showed peaks consistent with both small and mRNA species, while RNA was not detectable in EV preparations (Figures S2A,B in Supplementary Material). Overall, these data demonstrate that EVs found in healthy human plasma bias purified monocytes to a pro-inflammatory, M1 phenotype.

### EV Cell of Origin Determines Effect of EVs on Monocytes

**Figure 3:**
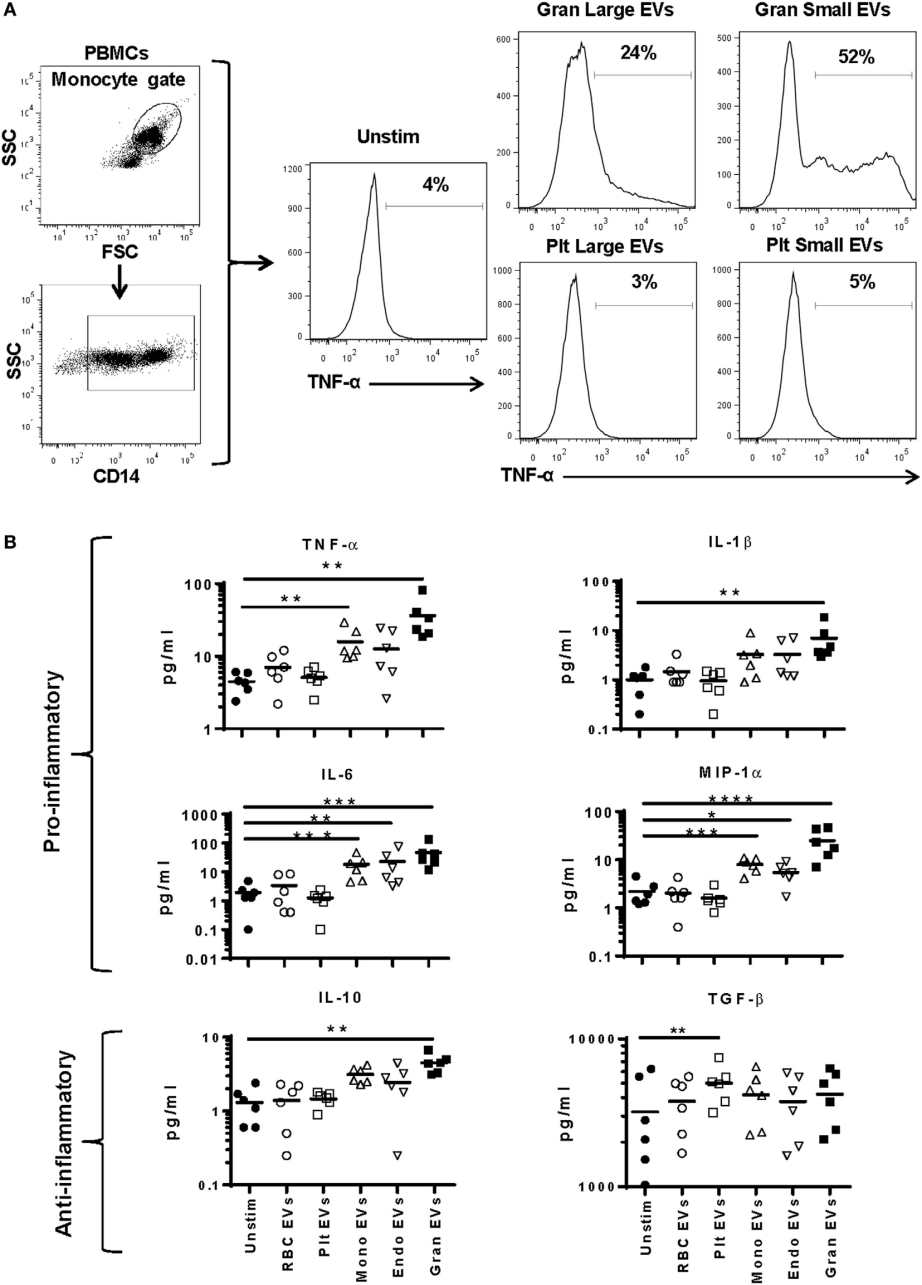

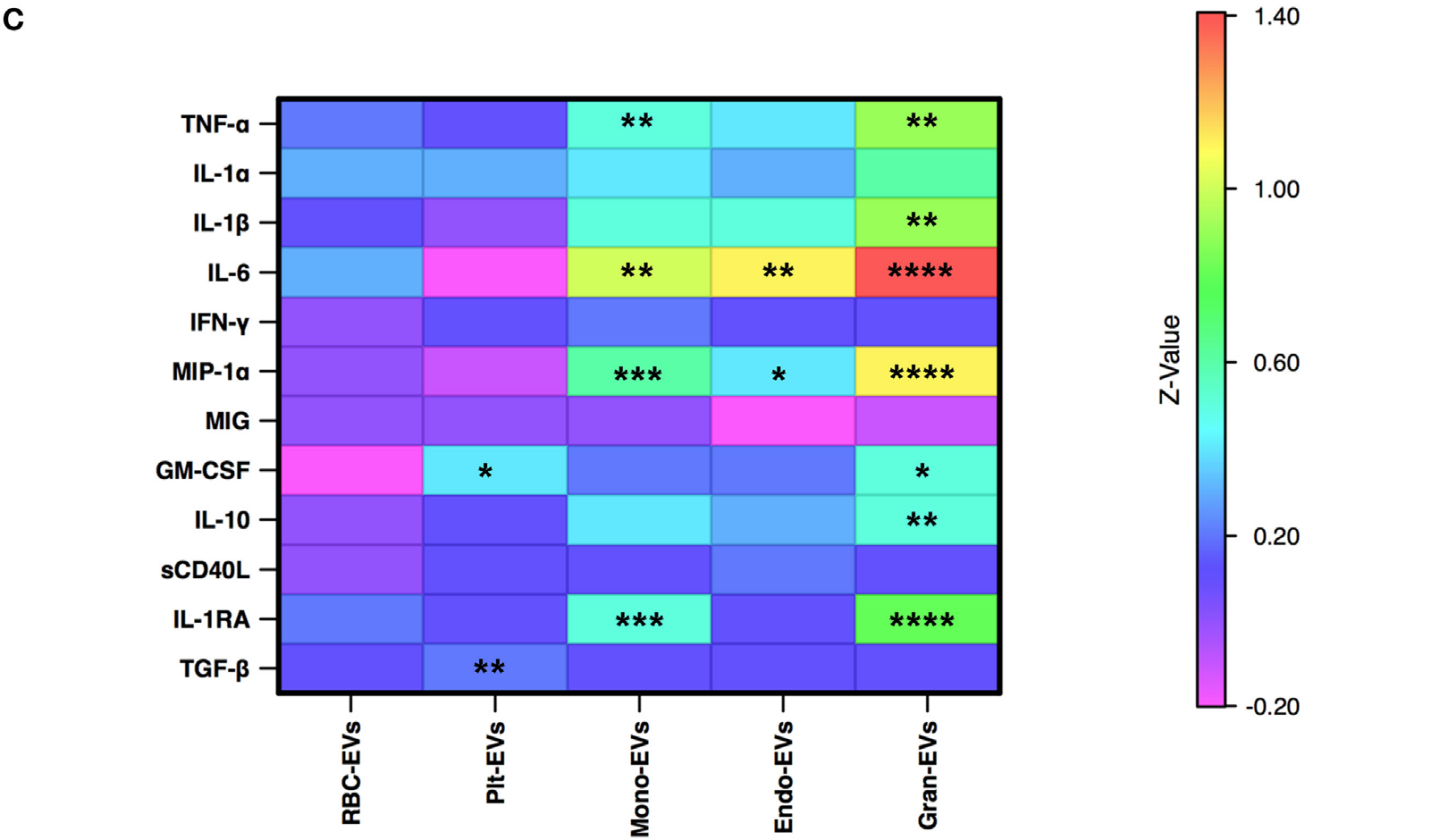
Cytokine secretion by monocytes stimulated with subtypes of extracellular vesicles (EVs). Monocytes from peripheral blood mononuclear cells of six healthy donors were purified by negative selection. Six replicates of red blood cell-, platelet-, monocyte-, endothelial-, and granulocyte-EVs were prepared as described in the “Materials and Methods” section. Monocytes were cultured unstimulated or incubated with noted EV subtypes for 24 h. **(A)** Two independent experiments were run with small (enriched for exosomes) and large (enriched for MVs) fractions of granulocyte- and platelet-EVs, and the percentage of monocytes that produced TNF-α was measured by intracellular staining. Representative data showing intracellular cytokine staining of monocytes incubated with small and large fractions of granulocyte- and platelet-EVs. **(B)** Supernatants were collected at 24 h and were tested using a multiplex cytokine assay for 12 cytokines. Data were analyzed by ANOVA, and each condition was compared with the control condition using a Dunnett’s post-test. Data are shown for 6 of the 12 cytokines tested. **(C)** The log_10_ ratio of cytokines induced by incubating monocytes with five subtypes of EVs over the control condition is summarized in a heat map for all 12 cytokines (*p < 0.05, **p < 0.01, and ***p < 0.001).

To better discriminate which EVs in plasma affect monocyte phenotype, we tested EVs derived from purified cell populations. Monocytes were stimulated with smaller EVs (<220 nm, enriched for exosomes) and larger EVs (>220 nm, enriched for microvesicles) derived from granulocyte- and platelet-EVs for 16 h and monitored for TNF-α production. While both small and large EV fractions of granulocyte-EVs led to production of TNF-α, neither the small nor the large EV fractions of platelet-EVs induced the production of this pro-inflammatory cytokine (Figure 3A).

To more comprehensively measure EV subtype effects, purified monocytes were stimulated with RBC-, platelet-, monocyte-, endothelial-, or granulocyte-EVs, and a panel of 12 cytokines was measured in cell culture supernatants. Granulocyte-EVs were the most pro-inflammatory, inducing significant increases in monocyte secretion of TNF-α, IL-1β, IL-6, MIP-1α, and GM-CSF (Figure 3B). Stimulation of monocytes with monocyte-EVs led to a significant increase in TNF-α, IL-6, and MIP-1α, and endothelial-EVs induced IL-6 and MIP-1α secretion. Stimulation of monocytes with platelet-EVs led to a significant increase of TGF-β production. Analysis of all 12 cytokines revealed EV-induced changes in IL-10 and IL-1RA as well. Stimulation of monocytes with RBC-EVs did not change the secretion of cytokines that we measured (Figure 3C). In general, culturing monocytes with pure granulocyte-, monocyte-, and endothelial-EVs induced the secretion of several pro-inflammatory cytokines, in contrast to platelet- and RBC-EVs.

### Gene Regulation and Cell Surface Expression of Scavenger Receptors

The role of scavenger receptors in EV uptake is an area of active investigation (25, 35, 36). The mRNA expression of 24 scavenger receptors was measured by RNA-Seq to determine if these receptors are regulated in monocytes after plasma-EV exposure. The mRNA expression of scavenger receptors *SRA-1* (*CD20*4), *SRI-1* (*CD163*), *CD280, SRI-2* (*CD163L1*), *SRB-2* (*CD36*), and *SRJ-1* was downregulated, and the mRNA expression of *SRF-1*, and *CD209* was upregulated compared with the time-matched unstimulated condition (Figure S3A in Supplementary Material).

Several scavenger receptors were selected for analysis of protein expression and requirement for EV–monocyte binding. It has been shown that the scavenger receptor CD36 plays a role in thrombosis in mice by binding to endothelial-EVs (25). Monocytes express the scavenger receptor CD163, which binds to hemoglobin and haptoglobin-hemoglobin complex (37). As RBC-EVs are loaded with hemoglobin (38), we thought CD163 may play a role in EV binding. The mRNA expression of *CD206*, a receptor on monocytes that binds to mannose residues on bacteria (39), did not change in the RNA-Seq data, so this gene was included as a control (Figure S3B in Supplementary Material). To validate the mRNA findings, the cell surface expression of these receptors was determined. CD36 expression was decreased significantly 3 h after stimulation of monocytes with plasma-EVs. Expression of CD163 on the monocyte cell surface was decreased significantly at 3 and 24 h. Incubation of monocytes with plasma-EVs did not significantly change surface CD206 expression (Figure S3C in Supplementary Material). The surface expression of the scavenger receptors on monocytes was largely consistent with the mRNA expression data, with decreases seen after incubation with EVs.

### Gene Regulation and Cell Surface Expression of Toll-Like Receptors

The expression of *TLR1, TLR2*, and *TLR4* mRNA was examined after exposure to plasma-EVs, as TLRs have been proposed as potential receptors for EVs (17, 18). The mRNA expression of *TLR1* was downregulated at 3 h and upregulated at 24 h. The mRNA expression of *TLR2* was upregulated at 3 and 24 h time points. Expression of *TLR4* mRNA was downregulated at 3 h (Figure S3D in Supplementary Material). The surface expression of the TLR receptors was not concordant with the gene expression data. TLR1 surface expression did not differ after exposure to plasma-EVs, while TLR2 and TLR4 surface expression dropped at 3 h after EV exposure (Figure S3E in Supplementary Material).

### Dependence of EV–Monocyte Binding on Scavenger Receptors and Toll-Like Receptors

**Figure 4:**
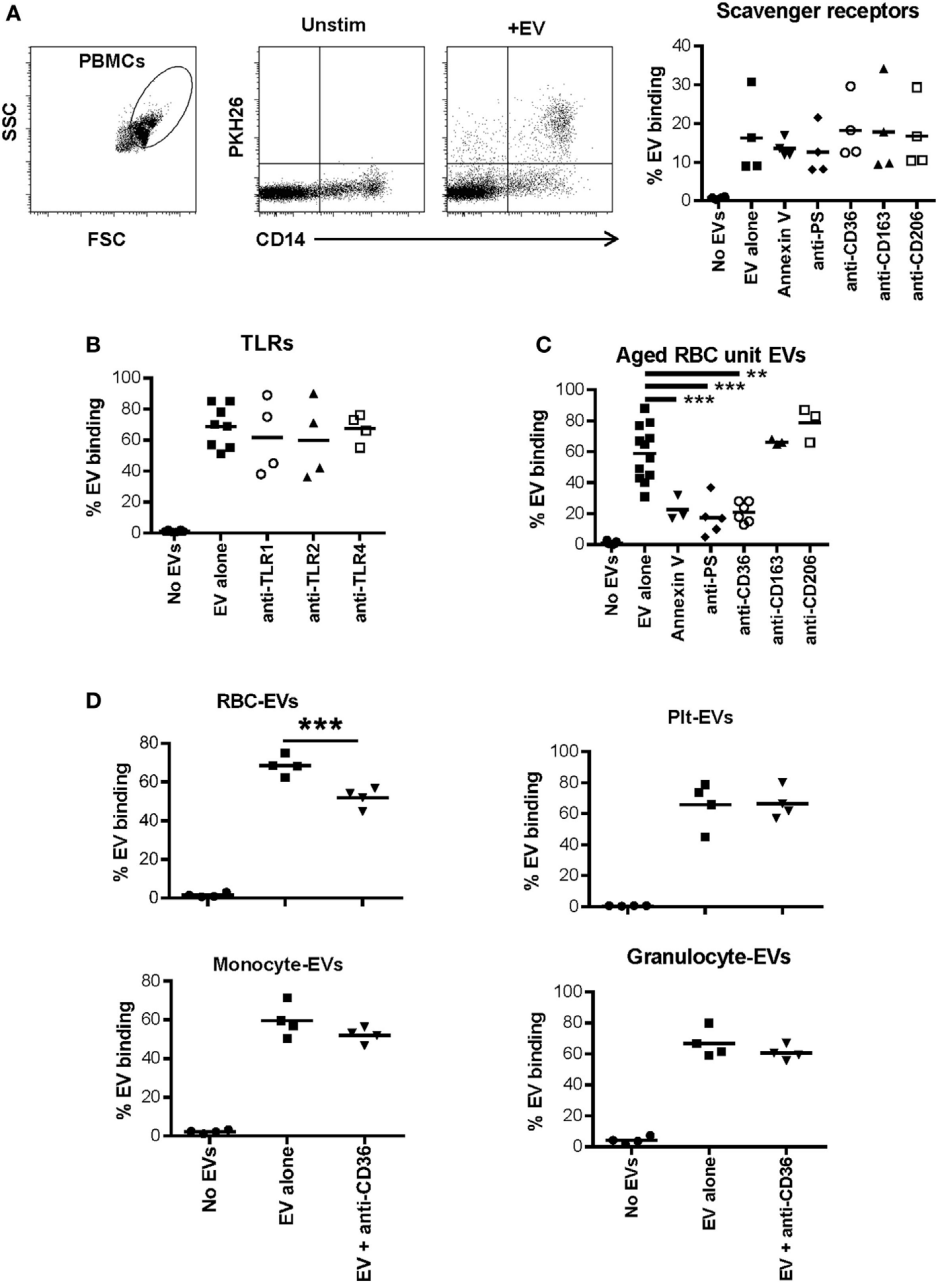
Role of scavenger receptors and TLRs in extracellular vesicle (EV)-monocyte binding. Peripheral blood mononuclear cells (PBMCs) (500,000) from 4 healthy donors were cultured unstimulated or incubated with 100 µL of PKH26 labeled EVs derived from plasma of 4 other healthy donors for 24 h. **(A)** PBMCs were incubated with EVs alone, or EVs pre-incubated with annexin V (1.0 µg/mL) or anti-phosphatidylserine (PS) antibody (1.0 µg/mL) for 1 h and added to PBMCs, or PBMCs were pre-incubated for 1 h with other antibodies noted on the *x*-axis at 1.0 µg/mL and added to PBMCs. After 24 h cells were stained with anti-CD14 and monocyte-EV binding was analyzed. **(B)** PBMCs were pre-incubated for 1 h with the noted TLR antagonists before incubation with EVs for 24 h as above. **(C)** PBMCs were incubated with EVs derived from red blood cell (RBC) units stored for 42 days, and binding inhibitors were added as above. **(D)** Binding of EVs derived from four different purified cell types to monocytes was assessed with or without pre-incubation of PBMCs with anti-CD36 antibody. EV binding inhibition conditions were compared with the EV alone condition by ANOVA with Dunnett’s post-test **(A–C)** or by *t*-test **(D)** (*p < 0.05, **p < 0.01, and ***p < 0.001).

Plasma-EVs were incubated with PBMCs for 24 h with or without the addition of annexin V or antibodies to phosphatidyl serine or several scavenger receptors. Blocking phosphatidyl serine, CD36, CD163, or CD206 did not affect EV–monocyte binding (Figure 4A). Similarly, blockade of TLR1, TLR2, or TLR4 had no effect on EV–monocyte binding (Figure 4B). To ensure that the TLR antibodies had blocking activity, PBMCs from two subjects were activated with LPS or Pam3CSK4 and incubated with TLR4 or TLR1/2 antibodies, respectively. Monocyte TNF-α production was decreased 30–80% by the TLR antibodies (Figure S4A in Supplementary Material).

In addition to testing the ability of plasma-EVs to bind to monocytes, EVs derived from packed RBC units stored for 42 days were examined for their binding capacity to monocytes. As we previously published, the predominant EV subtype in these preparations was RBC-derived (7). Incubation of RBC unit-EVs with annexin V or anti-phosphatidyl serine antibody before incubation with monocytes significantly reduced binding to monocytes. Incubation of monocytes with anti-CD36 antibody also significantly reduced binding to RBC unit-EVs (Figure 4C).

Based on the data that CD36 blockade decreased interaction of RBC unit- but not plasma-EVs with monocytes, the binding capacity of, monocyte-, granulocyte-, RBC-, and platelet-EVs to primary monocytes was tested with or without monoclonal antibody blockade of scavenger receptor CD36. A representative titration of antibody is shown for purified RBC-EVs and monocytes (Figure S4B in Supplementary Material). Inhibition of binding of platelet-, granulocyte-, and monocyte-EVs to primary monocytes in PBMC cultures was tested, and none showed significant inhibition after incubation with anti-CD36 antibody. These data show that CD36 is important in the binding of RBC-EVs to monocytes, but not for EVs derived from platelets, granulocytes, or monocytes (Figure 4D).

### Cellular Origin of EVs in Healthy Subjects

**Figure 5:**
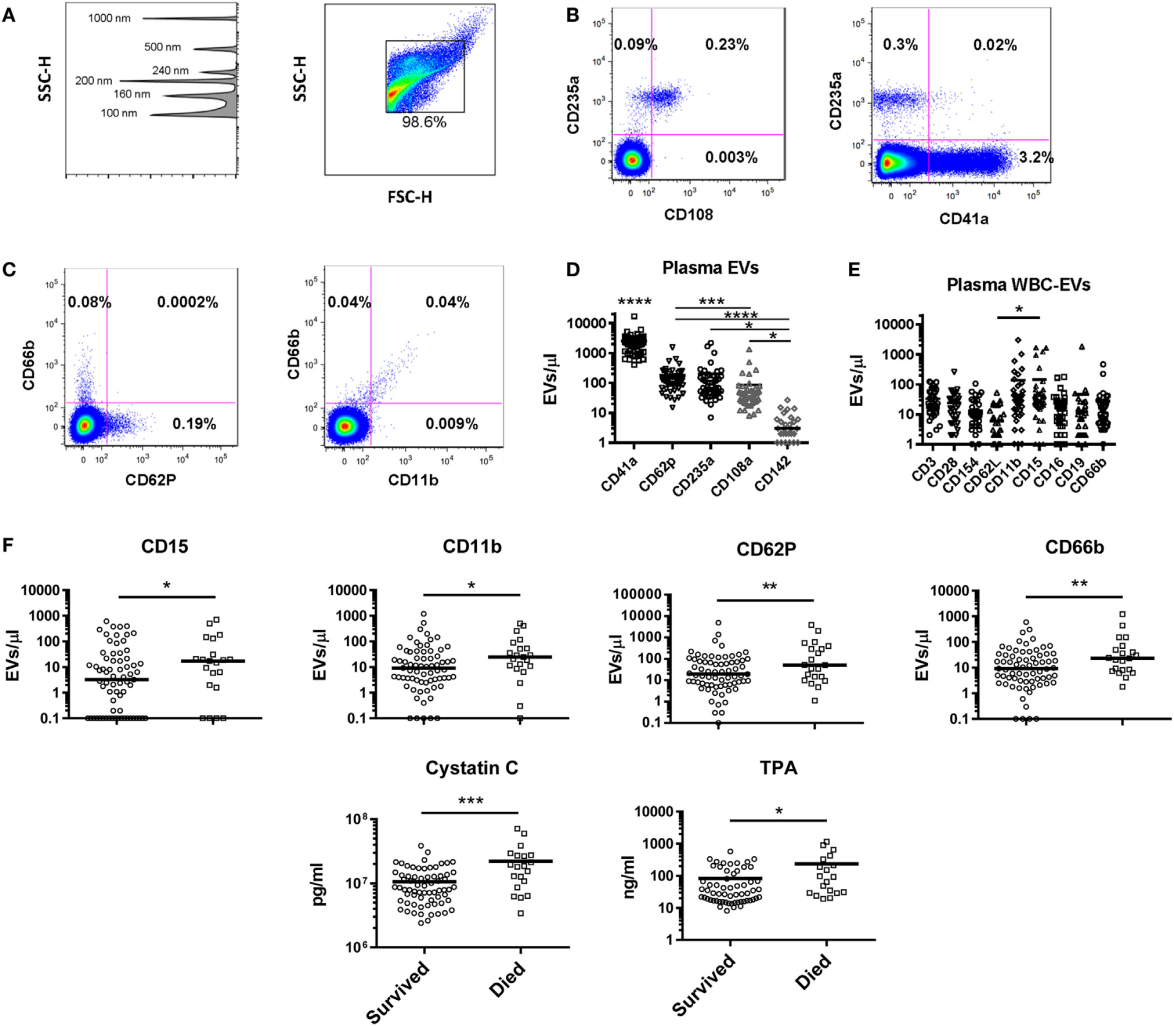
Extracellular vesicles (EVs) in healthy controls and as predictors of mortality in intensive care unit patients. **(A)** Gating strategy for EVs shows detection of beads sized 100–1,000 nm on the SSC channel in the left panel, and gating of EVs in the right panel. Representative flow cytometry plots for **(B)** red blood cell (RBC) and **(C)** granulocyte markers are shown. **(D)** Levels of EVs were measured in 48 healthy control subjects using a panel of markers to identify EVs derived from platelets (CD41a and CD62P), RBCs (CD235a and CD108a), and endothelial cells (CD142). CD41a^+^ EVs were significantly more abundant than all other populations (*p* < 0.0001); all other significant differences are noted on the graph. **(E)** White blood cell (WBC)-EVs were characterized based on cell of origin (CD3, CD16, CD19, CD66b) and expression of co-stimulatory (CD28 and CD154), and adhesion molecules (CD62L, CD11b, and CD15). **(F)** Of 62 parameters measured at baseline in 100 critically ill subjects, the 6 associated with 28-day mortality are shown, including EVs expressing four markers (CD15, CD11b, CD62P, and CD66b) (*p < 0.05, **p < 0.01, ***p < 0.001, and ****p < 0.0001).

The plasma EV profile in 48 healthy subjects (42% male, median age 46) was examined using a panel of markers to identify EVs bearing markers of endothelial cells (CD142, CD62P), platelets (CD41a, CD62P), RBCs (CD235a, CD108a), and multiple WBC populations. Flow plots gated on EVs (events <1 μm by forward and side scatter, Figure 5A) revealed that the RBC marker CD235a and platelet marker CD41a were found on separate populations, while the RBC activation marker CD108a was only found on EVs also positive for CD235a (Figure 5B). Similarly, EVs bearing the granulocyte marker CD66b were a separate population from those bearing CD62P (P-selectin), while those bearing the adhesion molecule CD11b were found almost exclusively on the granulocyte-EVs (Figure 5C). Platelet-EVs were more numerous than those derived from any other cell type measured, and those bearing CD142 (tissue factor) were rarely detected (Figure 5D). To characterize WBC-EVs, various cell lineage and activation markers were examined (Figure 5E). EVs bearing markers of T cells (CD3), B cells (CD19), monocyte/NK cells (CD16), and granulocytes (CD66b) were all detected. The adhesion molecule CD15 was detected at higher levels than the adhesion molecule CD62L (L-selectin), though overall there were insignificant differences in expression of various WBC markers on EVs in healthy subjects. These data demonstrate that EVs from different cell subtypes can be distinguished by flow cytometry and that WBC-EVs are present at low-level in healthy subjects’ plasma.

### Predictors of Mortality in Critically Ill Patients

As part of the ABLE study to analyze the effect of RBC unit storage on clinical outcomes in transfused, critically ill patients (30), serial samples were collected from 100 subjects randomized to receive RBC units that had been stored for a shorter or longer period. These subjects were 95% medical admissions, were 50% female, and had a median age of 67. These samples were tested for an array of 16 coagulation, 27 cytokine, 3 immune cell, and 14 EV markers. The ABLE study found no effect of RBC unit storage age on mortality or other clinical outcomes, and the longitudinal analysis of the relationship of these markers with RBC unit age is the subject of a manuscript in preparation. For this study, we analyzed the 100 subjects in aggregate to determine if immune or coagulation markers in samples collected at ICU admission, pre-transfusion predicted subsequent 28-day mortality, which was 22% in these subjects (Table 1). Elevated levels of six factors upon intensive care unit (ICU) admission were associated with subsequent mortality: cystatin C, TPA, and EVs bearing the markers CD15, CD11b, CD62P, or CD66b (Figure 5F).

**Table 1:** Baseline predictors of mortality.

Coagulation			Cytokines			Extracellular vesicles		
Parameter	*p* Value	HR (cb)	Parameter	*p* Value	HR (cb)	Parameter	*p* Value	HR (cb)
PT	0.33	1.02 (0.97–1.08)	GM-CSF	0.059	0.70 (0.48–1.01)	EV concentration	0.83	1.04 (0.74–1.45)
PTT	0.085	1.01 (0.999–1.017)	IFN-γ	0.84	0.97 (0.73–1.29)	Annexin V	0.85	0.98 (0.80–1.20)
D-dimer	0.7	1.04 (0.86–1.25)	IL-10	0.84	1.03 (0.77–1.37)	CD3	0.78	1.04 (0.79–1.37)
Factor II	0.28	0.99 (0.97–1.01)	IL-12p70	0.84	1.06 (0.58–1.96)	CD14	0.56	1.06 (0.88–1.27)
Factor V	0.21	0.99 (0.98–1.004)	IL-17A	0.3	0.81 (0.54–1.21)	CD16	0.62	0.93 (0.70–1.24)
Factor VII	0.28	0.75 (0.45–1.26)	IL-1β	0.89	1.06 (0.45–2.49)	CD19	0.8	0.97 (0.76–1.24)
Factor VIII40	0.63	1.00 (0.998–1.003)	IL-2	0.62	0.85 (0.45–1.61)	CD28	0.22	1.11 (0.94–1.31)
Factor IX	0.96	1.00 (0.99–1.01)	IL-21	0.31	0.73 (0.40–1.34)	CD152	0.59	0.93 (0.72–1.21)
Factor X	0.95	0.99 (0.98–1.01)	IL-23	0.84	0.98 (0.78–1.22)	CD41a	0.32	1.12 (0.89–1.41)
ATIII	0.74	1.03 (0.88–1.20)	IL-6	0.62	0.92 (0.67–1.27)	CD62L	0.091	1.22 (0.97–1.55)
PC	0.18	0.90 (0.78–1.05)	IL-7	0.69	1.01 (0.94–1.09)	CD108a	0.71	0.94 (0.70–1.28)
FIB	0.26	0.99 (0.98–1.01)	IL-8	0.62	1.10 (0.75–1.61)	CD235a	0.98	1.00 (0.76–1.31)
TM	0.69	1.18 (0.51–2.73)	ITAC	0.92	0.98 (0.70–1.38)	CD11b	**0.01**	1.44 (1.09–1.91)
ECPR	0.72	1.01 (0.94–1.10)	MIP-1α	0.19	0.73 (0.46–1.17)	CD15	**0.021**	1.25 (1.03–1.52)
TPA	**0.011**	1.57 (1.10–2.22)	MIP-1β	0.24	0.70 (0.38–1.26)	CD62P	**0.008**	1.34 (1.08–1.66)
PAI-1	0.27	1.14 (0.93–1.44)	TNF-α	0.81	1.06 (0.67–1.67)	CD66b	**0.001**	1.60 (1.20–2.15)
			EGF	0.82	1.03 (0.77–1.39)			
			FGF	0.82	0.97 (0.72–1.30)	*Cellular immunity*		
			VEGF	0.29	1.10 (0.92–1.31)	Treg	0.098	1.25 (0.96–1.63)
			β2-Microglobulin	0.28	1.12 (0.91–1.31)	CD4-IL-7	0.21	2.30 (0.63–8.37)
			Cystatin C	**<0.0001**	1.04 (1.02–1.07)	CD8-IFN-γ	0.3	0.98 (0.94–1.02)
			MPO	0.26	1.19 (0.88–1.59)			
			PDFG AB/BB	0.59	1.05 (0.88–1.26)			
			RANTES	0.47	0.91 (0.70–1.18)			
			sICAM-1	0.57	1.01 (0.98–1.04)			
			sVCAM-1	0.084	1.02 (1.00–1.04)			

*HR, hazard ratio; cb, confidence bound; PAI-1, plasminogen activator inhibitor type-1; TPA, tissue plasminogen activator; EV, extracellular vesicle*.

*Significant values in bold*.

*HRs reflect the change in the hazard that is associated with one unit of change in particular variables except as noted below*.

*10 units of change: GM-CSF, IFN-γ, IL-10, IL-23, IL-6, IL-8, ITAC, EGF, FGF, and VEGF*.

*100 units of change: PDFG AA/BB, CD3, CD14, CD16, CD19, CD28, CD152, CD62L, CD108a, CD11b, CD15, and CD66b*.

*1,000 units of change: RANTES, CD235a, CD62P, and annexin V*.

*10,000 units of change: MPO, sICAM-1, CD41a, and EV concentration*.

*1 million units of change: cystatin C*.

*10 million units of change: β2-microglobulin*.

## Discussion

In this article, we show that plasma-EVs biased primary monocytes toward an M1 profile and led to secretion of inflammatory mediators. Inspection of EVs from purified cell populations revealed that monocyte-, granulocyte-, and endothelial-EVs drove a pro-inflammatory monocyte response, with granulocyte-EVs inducing the broadest and highest magnitude response. In addition, platelet-EVs were the only population to induce monocyte production of the anti-inflammatory cytokine TGF-β, and RBC-EVs did not regulate cytokines and chemokines that we measured. The scavenger receptor CD36 is a potential receptor for RBC-EVs but not for the other EV subtypes tested. Finally, we showed that CD66b^+^ granulocyte-EVs are early predictors of mortality in ICU patients.

Knowing that plasma-EVs are comprised of a heterogeneous mix of subtypes derived from distinct cells of origin (40–42), and to better understand which EVs in plasma bias monocytes to a pro-inflammatory phenotype, EVs were prepared from purified cell populations using routine procedures in a blood bank setting (RBC- and platelet-EVs), or by culturing unstimulated cells (monocyte-, granulocyte-, and endothelial-EVs) to avoid the effect of mitogens on EV cargo (43). Granulocyte-EVs were found to be the most potent pro-inflammatory agents of the EV subtypes studied, followed by monocyte- and endothelial-EVs in pro-inflammatory activity in our in vitro assays. Granulocyte-EVs have also been described to augment or suppress immune response (42, 43). Eken et al. have reported that EVs released by granulocytes induce a MerTK-dependent anti-inflammatory pathway in monocyte-derived macrophages (44). One difference between that study and the current work is that they stimulated purified granulocytes with fMLP to generate EVs. This stimulation could have affected the EV cargo and composition compared with EVs that are released during physiologic conditions or from granulocytes that were not stimulated (45). In contrast to the pro-inflammatory EVs, platelet-EVs induced TGF-β secretion by monocytes. TGF-β is a multifunctional cytokine with a dominant immunosuppressive activity. While it plays a positive role in tissue repair and in the control of autoimmune and infectious diseases, its upregulation may increase the growth of tumor cells (46). Platelet-EVs may have therapeutic value in tissue repair and downregulating the immune system in autoimmune diseases.

After demonstrating that plasma-EVs activated monocytes, we searched for antibodies that could block EV–monocyte binding. The RNA-Seq data showed the downregulation of a cluster of scavenger receptors on monocytes followed by their stimulation with total EVs. Two well-known scavenger receptors that were downregulated (*CD36* and *CD163*) and one whose gene transcription did not change (*CD206*) were selected for functional studies. Blockade of these receptors did not affect the binding of plasma-EVs to monocytes, though anti-CD36 antibody blocked RBC-EV binding to monocytes. A prior study has shown that endothelial-EVs bind to the scavenger receptor CD36 on platelets and contribute to thrombosis in mice (25). We found that the scavenger receptors CD36 and CD163 were downregulated on the cell surface of monocytes stimulated with plasma-EVs; however, a binding inhibition assay showed that only CD36 was involved in RBC-EV binding to monocytes. Given the relative lack of effect of RBC-EVs in our monocyte stimulation assays, it is unclear how effective CD36 blockade would be in regulating immune changes potentially induced by transfusions rich in RBC-EVs, though other investigators have described RBC-EVs as having immune suppressive activity (9). Blockade of CD9, CD81, CD54, CD11a, CD51, and CD61 is reported to reduce EV uptake by dendritic cells (22). The interaction of lectin family members such as CD205 and CD209 has also been studied, and their blockade leads to reduced EV uptake (21, 23). In general, EV uptake cannot be prevented completely by blockade of one receptor, suggesting that several receptors are involved (12, 19, 21–24). TLRs have also been described to play a role in EV uptake. We tested antibodies against TLR1, TLR2, and TLR4, and they did not block EV–monocyte binding. It has been shown that tumor-exosomes control the expansion of myeloid-derived suppressor cells through TLR2 and not TLR4, which leads to secretion of IL-6 and suppression of CD8^+^ T cells (18). However, it was found in another study that stimulation of the monocytic cell line THP1 with tumor-exosomes induced TNF-α, IL-1β, and IL-6 through TLR2 and TLR4 signaling (17).

**Figure 6:**
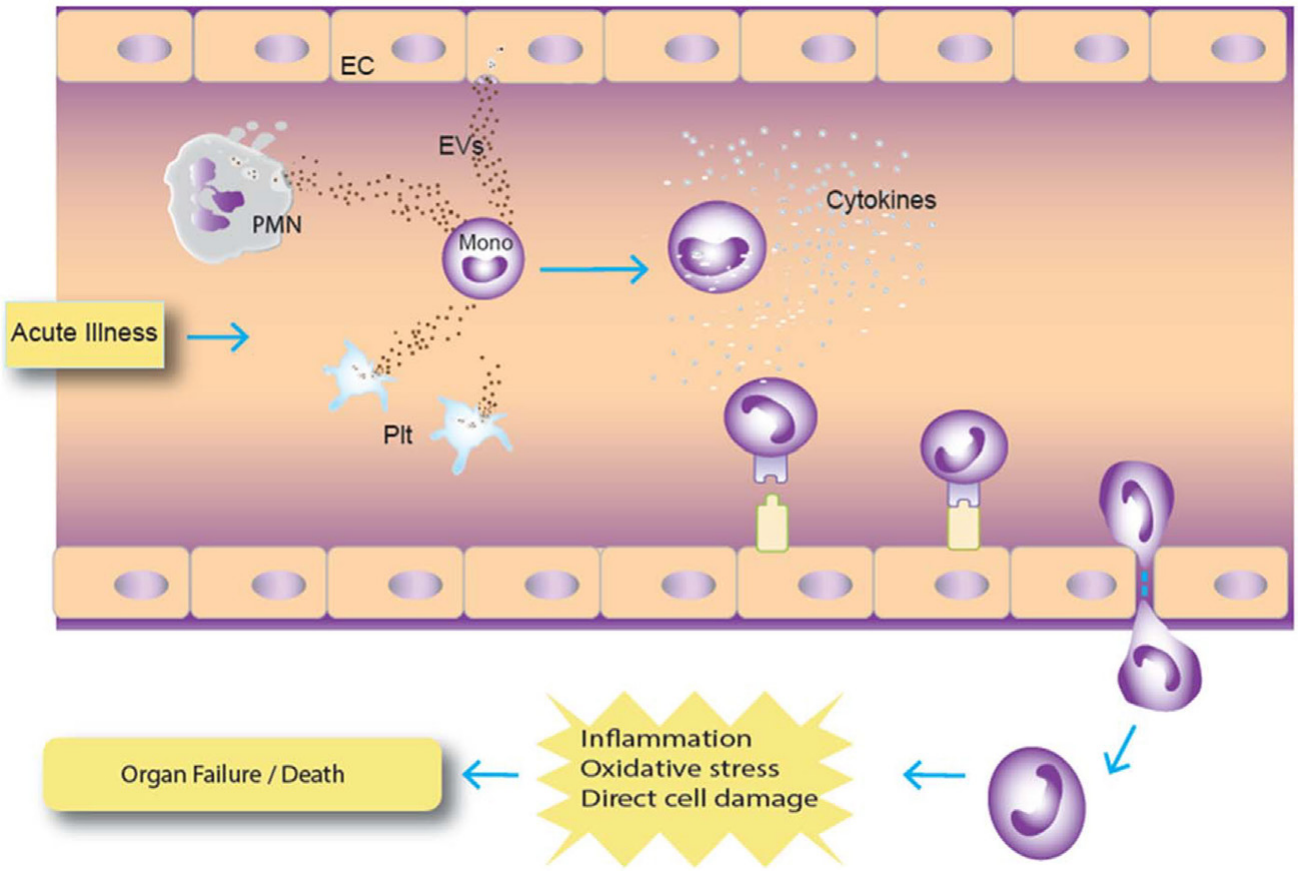
Hypothetical model of extracellular vesicle (EV) interaction with monocytes. In this model, acute infection results in augmented release of EVs from cells, particularly granulocytes, which are ingested by monocytes. Activated monocytes can then differentiate and migrate to tissues, which can increase tissue inflammation and damage. Abbreviations: PMN, polymorphonuclear cell (granulocyte); Plt, platelet.

In our *ex vivo* study on plasma samples from ICU patients, a panel of 43 cytokines and coagulation factors was examined, and only TPA and cystatin C were associated with mortality risk (47–52). TPA is involved with fibrinolysis and has been reported to be a marker for subsequent mortality in subjects hospitalized for acute dyspnea (50). Its significant release by endothelial cells after traumatic injury has been shown to result in excessive bleeding and hyperfibrinolysis, which are known risk factors for mortality (48, 49). Cystatin C is a cysteine protease inhibitor that plays a role in catabolism of proteins, and it has been widely described as being associated with mortality risk in various disease states (47, 51, 52). In addition, 4 of the 14 EV markers tested were asso-ciated with increased death risk in this population of critically ill patients who primarily presented with medical as opposed to surgical diagnoses (30). The existing literature is relatively scarce describing the prognostic potential of EVs as predictors of mortality. Our results indicate that CD66b, CD15, CD11b, and CD62P EVs were predictors of mortality in our cohort of patients. Of these, CD66b is the most specific and is a marker for granulocytes (53). CD15 is expressed on granulocytes and monocytes (54), and given that levels of the monocyte markers CD14 and CD16 were not associated with risk of death, it is likely that the CD15 EVs arose from granulocytes rather than monocytes. CD11b is expressed on many cell types including granulocytes, monocytes, and lymphocytes (55), so while elevated CD11b-expressing EVs is consistent with a granulocyte cell of origin, this marker is not specific. CD62P is an endothelial and platelet marker (56), and in this study EVs bearing the platelet marker CD41a were not associated with mortality risk. CD41a-expressing EVs have been reported to be positively or negatively associated with mortality in prior studies (57–59). Endothelial-EV markers were not measured in this study, though it is possible that CD62P EVs were derived from endothelial cells. The upregulation of endothelial-EVs has been described in patients with systemic inflammation due to sickle cell disease (27). Given the exploratory nature of the large panel of analytes studied, correlations were not corrected for multiple comparisons. The pattern of predominantly granulocyte-EVs correlating with mortality risk is suggestive that the correlations were not random, though future studies will be needed to confirm the association. Of note, WBC- and granulocyte-EVs were recently reported to be associated with subsequent mortality in a population of critically ill burn patients, increasing confidence in the validity of the association (29). Our data are consistent with a model in which EVs from various cell types signal circulating monocytes, which can synthesize these signals to become activated and potentially participate in tissue damage (Figure 6).

Here, we have shown by gene expression analysis that incubation of monocytes with EVs polarized monocytes toward a pro-inflammatory state. Of five EV subtypes that were tested, monocyte-, endothelial-, and granulocyte-EVs induced production of pro-inflammatory cytokines in monocytes, and granulocyte-EVs were the most potent inflammation trigger. Platelet-EVs induced production of the anti-inflammatory cytokine TGF-β and GM-CSF, and RBC-EVs did not regulate cytokines and chemokines that were measured. We demonstrated a role for the scavenger receptor CD36 in the binding of RBC-EVs to monocytes. Finally, we have shown granulocyte-EVs (expressing CD66b) were early predictors of mortality in ICU patients. Characterization of anti-inflammatory subtypes of EVs may have therapeutic applications in inflammatory diseases including critical illness, and pro-inflammatory EVs could potentially be harnessed as vaccine adjuvants or targeted for blockade to reduce inflammation during critical illness.

## Ethics Statement

This study was carried out in accordance with the recommendations of the University of California, San Francisco Institutional Review Board with written informed consent from all subjects. All subjects gave written informed consent in accordance with the Declaration of Helsinki. The protocol was approved by the University of California, San Francisco Institutional Review Board.

## Author Contributions

AD designed, performed, and analyzed the *in vitro* experiments, designed the EV panels for patient samples, and wrote the manuscript. HI, RV, SK, JH, PS, and MC designed and/or performed analysis of patient samples. JL and PS provided clinical samples. MA-M, EJ, and SP assisted with design of laboratory methods. XD, AA, and KS designed and performed statistical analyses. PN designed and oversaw the project and wrote the manuscript.

## Conflict of Interest Statement

The authors declare that the research was conducted in the absence of any commercial or financial relationships that could be construed as a potential conflict of interest. The reviewer AB and handling Editor declared their shared affiliation.
