# Supplementary material for: Granulocyte-Derived Extracellular Vesicles Activate Monocytes and Are Associated With Mortality in Intensive Care Unit Patients
Source: Front Immunol. 2018 May 8;9:956. doi: 10.3389/fimmu.2018.00956 (PMC5951932; doi:10.3389/fimmu.2018.00956)
Supplement: Supplementary file 1 [file Image_1.pdf]

Supplementary Figure 1

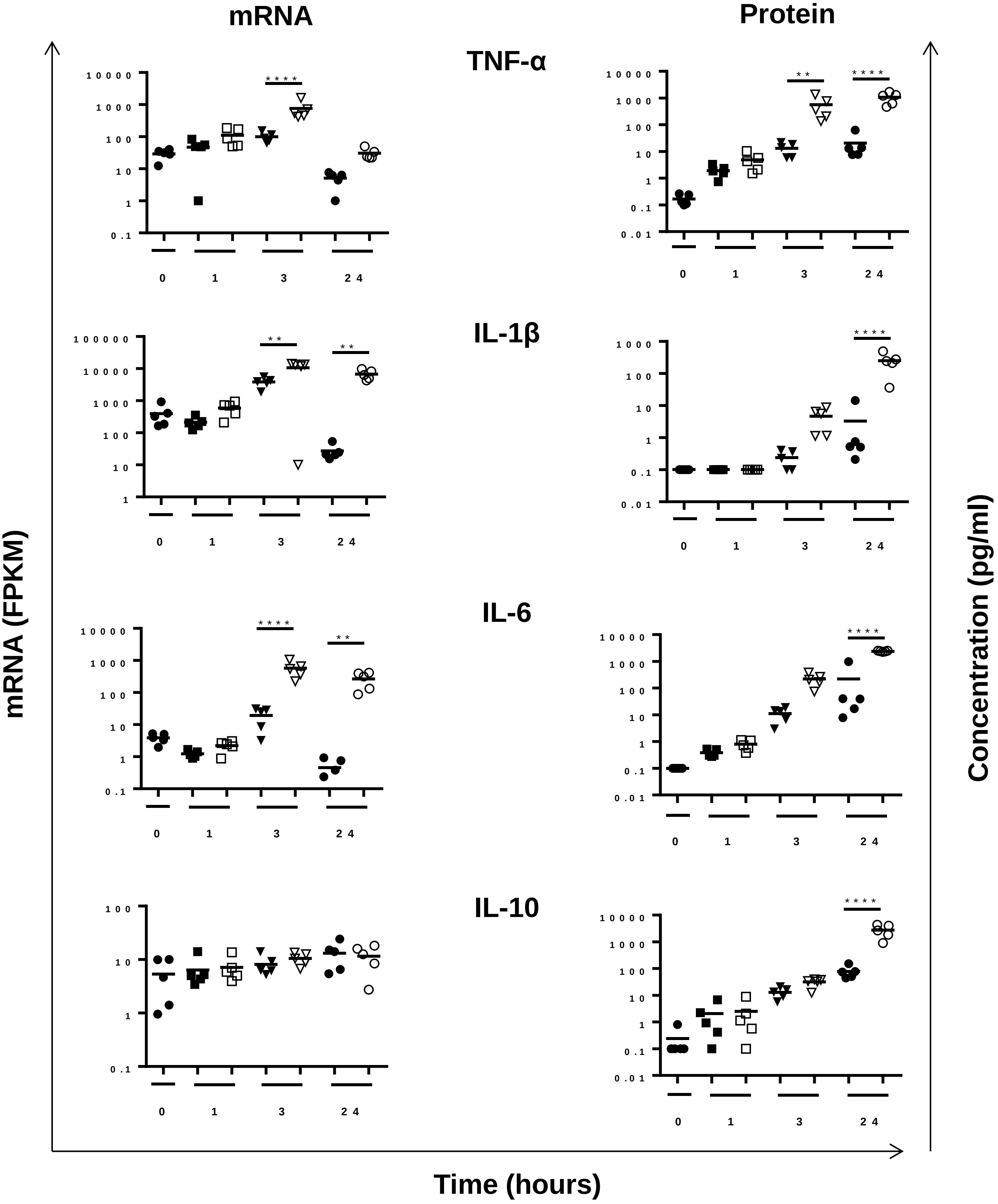

Supplementary Figure 1: mRNA and protein levels of cytokines induced in monocytes incubated with EVs. A) Gene expression detected by RNA-Seq and protein expression detected by multiplex assay were compared for 4 representative cytokines after EV-monocyte incubation. Filled symbols represent monocytes (10^6) cultured alone and empty symbols represent paired samples incubated with EVs (10^6). **p<0.01, ****p<0.0001

Supplementary Figure 2

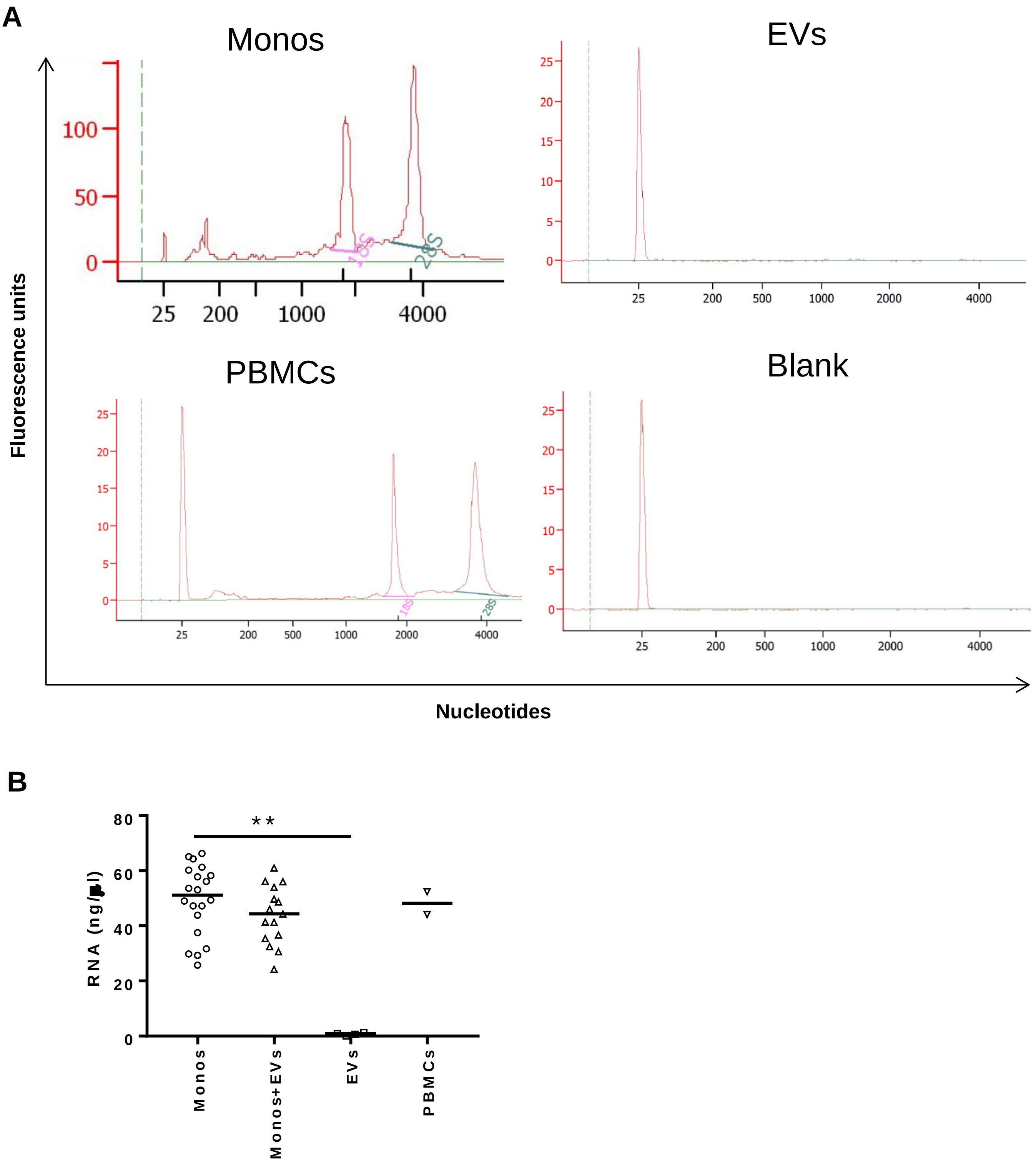

Supplementary Figure 2: RNA quality and content of monocyte and EV preparations. A) Representative electropherograms are shown for monocyte, EV, PBMC, and blank conditions. RNA was extracted from 2x10^{6} cells or EVs. An internal 25 nucleotide control was included in each assay. B) RNA content by Nanodrop for the monocyte conditions with or without EVs used for RNA-Seq experiments. In a separate experiment RNA was quantified for EVs and PBMC positive controls. **p<0.01

Supplementary Figure 3

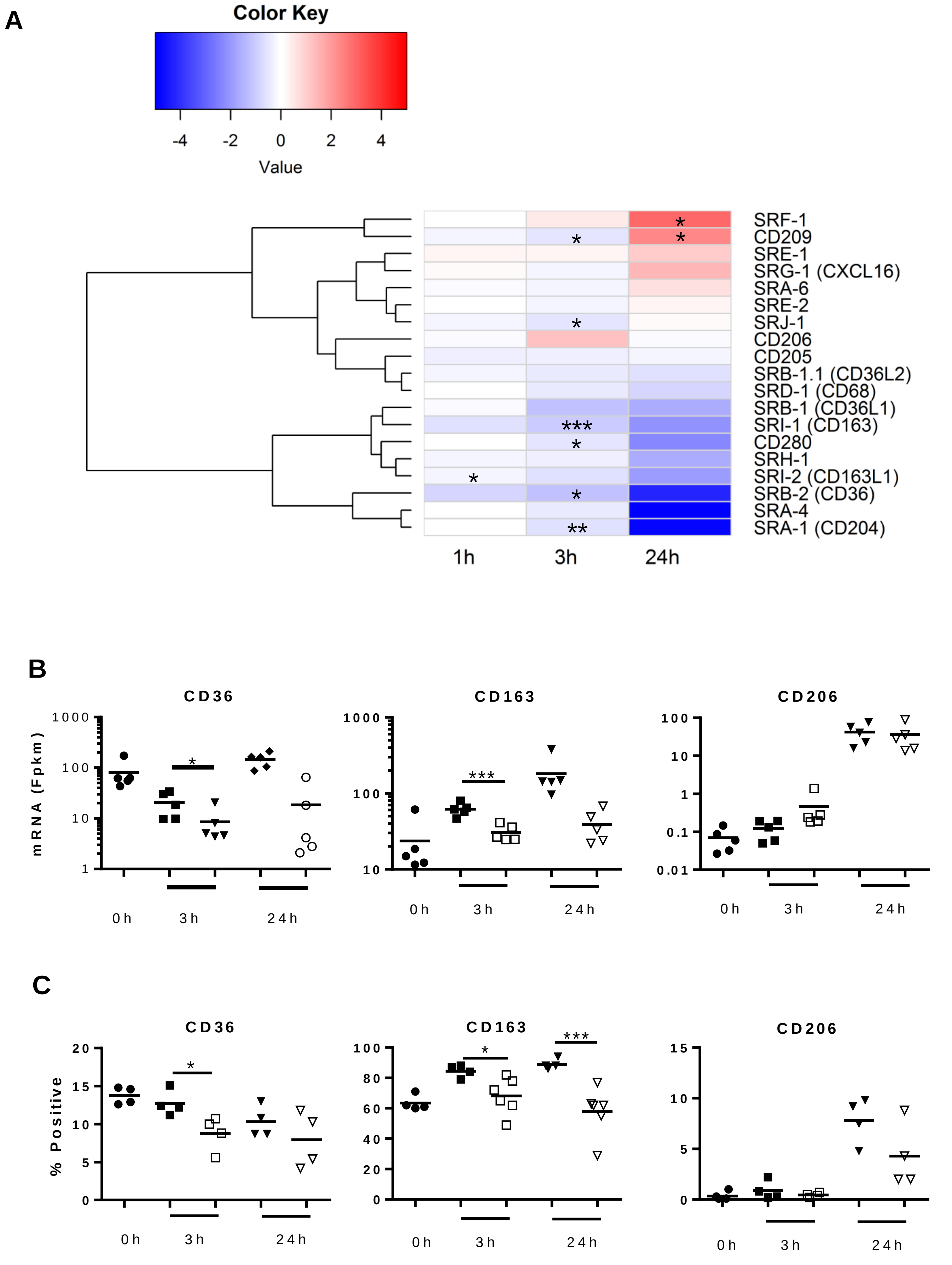

Supplementary Figure 3

D

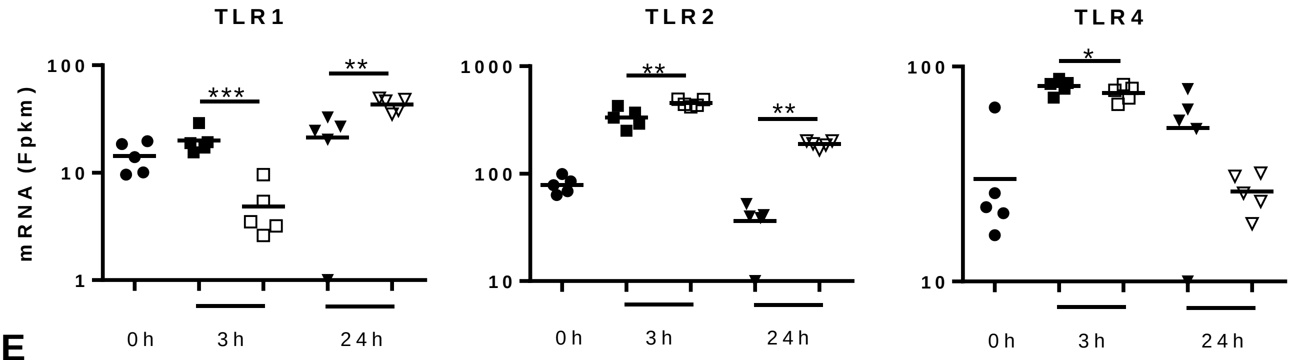

E

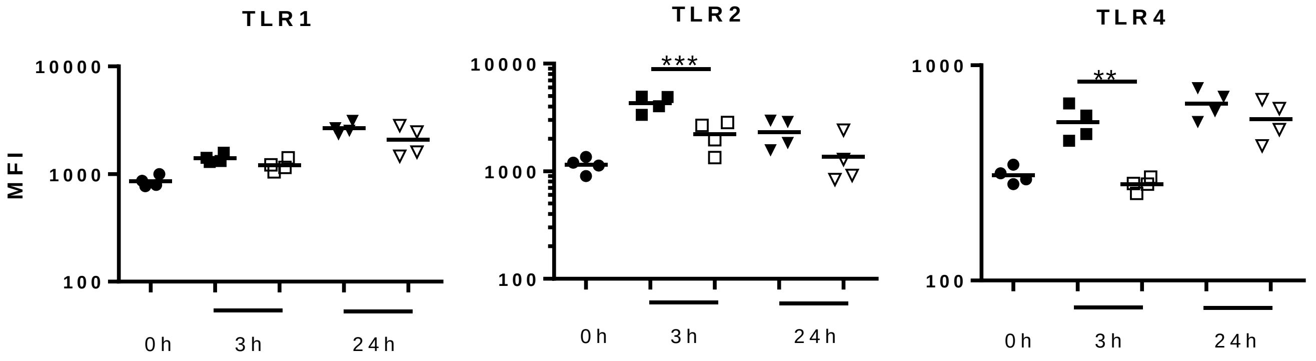

Supplementary Figure 3: Scavenger receptor modulation after monocyte incubation with EVs. A) Expression of scavenger receptor mRNA was measured in purified monocytes (10^6) from 5 subjects incubated with EVs (10^6) from 5 separate subjects. Data were $\log_2$ transformed and compared to the paired unstimulated monocyte condition as a ratio. Expression of mRNA for 2 receptors was upregulated and for 7 was downregulated at 3 and/or 24h. B) Individual data points for the mRNA expression data are shown for 3 scavenger receptors. C) Surface expression of 3 scavenger receptors was measured in a separate experiment using PBMCs from 4 subjects incubated with EVs from 4 separate subjects. D) Expression of TLR mRNA is shown from the same 5 subjects as above. E) Surface expression of TLR receptors was measured in a separate experiment using PBMCs from 4 subjects incubated with EVs from 4 separate subjects.
The level of TLR expression was assessed by flow cytometry after gating on CD14+ monocytes.
*$p<0.05$, **$p<0.01$, ***$p<0.001$

Supplementary Figure 4

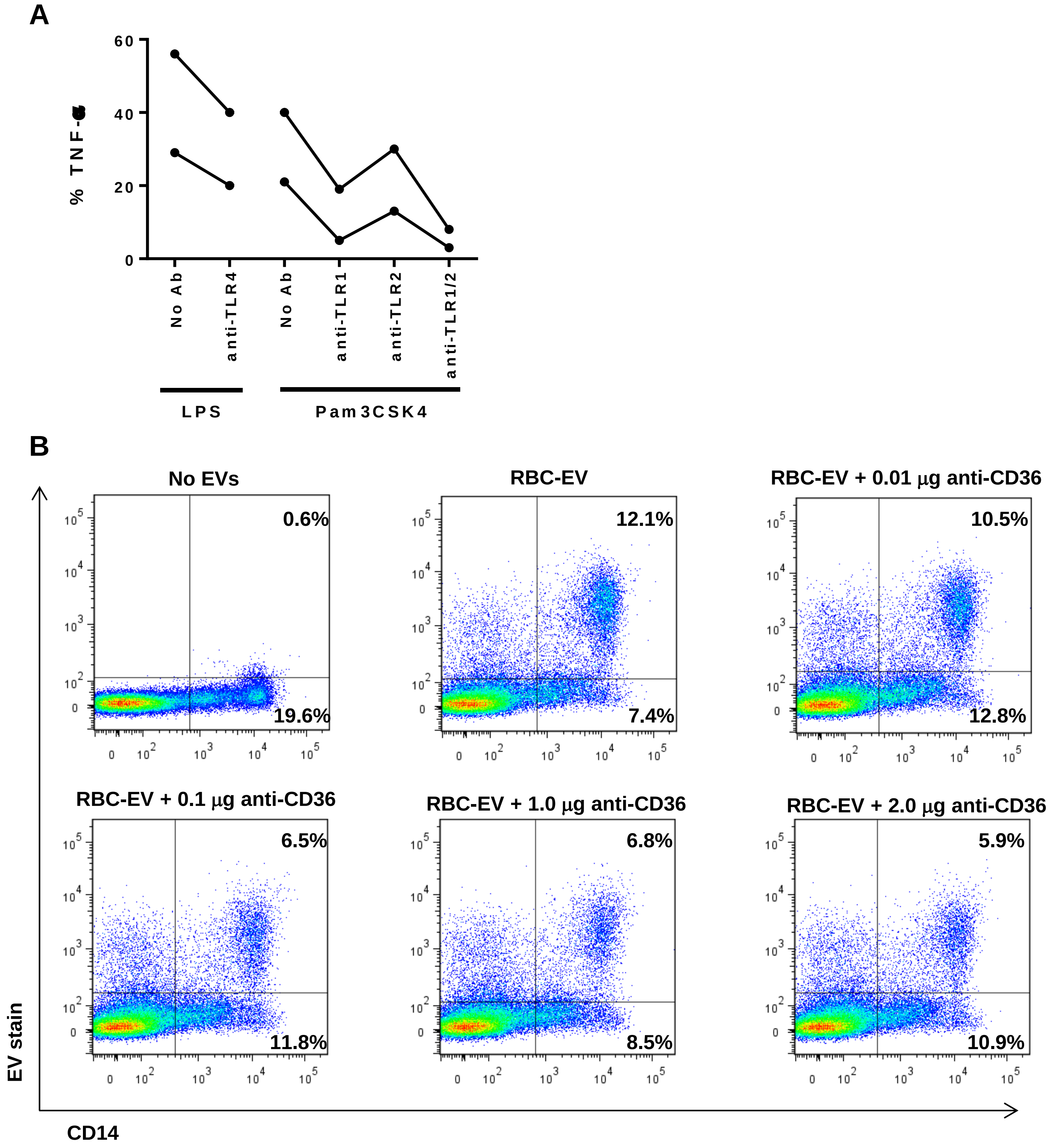

Supplementary Figure 4: TLR antagonist activity and CD36 antibody titration. A) To measure whether or not the commercially acquired TLR antibodies had blocking activity, the ability of each to suppress TNF-α production by monocytes was measured. PBMCs from 2 subjects were incubated with the indicated blocking antibody (1 μg/mL) for 1h, then stimulated with LPS (5 ng/mL) or Pam3CSK4 (20 ng/ml) for 16h. TNF-α production by monocytes was assessed by intracellular cytokine staining and flow cytometry. B) Individual dot plots show EV (stained with PKH26) uptake by monocytes (CD14+) for RBC-EVs incubated with monocytes pre-incubated for 1h with increasing concentrations of anti-CD36 antibody.
